# Acute toxicity of binary mixtures: alternative methods, QSAR and mechanisms

**DOI:** 10.2478/v10102-010-0025-z

**Published:** 2010-11

**Authors:** Miloň Tichý, Iveta Hanzlíková, Marián Rucki, Adéla Pokorná, Rút Uzlová, Jana Tumová

**Affiliations:** National Institute of Public Health, Praha, Czech Republic

Committee accepted the program of chemical safety in October 2003 called REACH, meaning “Registration, Evaluation, Authorization of Chemicals”. The protection of health of nature, including human beings, against harmful effects of chemicals is a goal of these programs. The development and research on new chemicals can become, however, cheaper than their testing and registration. The aim of effort of the present is, thus, to develop and to use alternative methods of testing toxic and adverse effects of chemicals, which would be cheaper and more informative than traditional tests with experimental animals.

Term “alternative tests” is used in toxicology to mark such tests, which can be used to substitute traditional tests with experimental animals and afford at least the same information, they are quicker, must be easy, cheaper, and giving information sufficient and comparable with information given by the traditional methods, or better. A joint term “integrated” means that more methods form a set of tests often quite different in their character (eg. biological, physicochemical and models). By this way we reach the situation when toxicity of chemical are found “without mice or rats” and to “chemistry without test-tubes” – to predictive toxicology. Terms “experimental toxicology” and methods/models *in silico*, determination by calculation, may look as an absurd connection. Experimetnal toxicology uses experimental animals, tissues or organs, cells, *in silico* methods use calculators. On the contrary in fact, they have a common goal: Methods of both types belong to alternative methods for determination of hazard of chemicals, i.e. toxic and adverse effects of chemicals. QSAR (Quantitative Structure – Activity Relationships) models are the most known and the best developed among the alternative methods *in silico*. These models/mathematical equations become usable even for legislative usage substituting other methods. OECD committee for validation of QSAR models has formed a set of rules for buiding such acceptable QSAR models.

QSAR is acronym for Quantitative Structure – Activity Relationships, for quantitative relationships between chemical structure and magnitude of biological effects of chemicals produced by chemical industry or in laboratories. Not of chemicals of natural origin when specific mechanisms of action participate. The QSAR models express a relationship between magnitude of biological effects (BA) and changes in a molecular a structure, eg. changes of substituents, in a series of chemicals (X) by a mathematical function (f). A series may be of homogeneous, a series of derivatives of benzene of the same basic structure, or of heterogeneous nature like miscellaneous chemicals of various structure:

$$\begin{array}{c} \text{B}{\text{A}}_{\text{i}}\text{=f(}{\text{X}}_{\text{i}}\text{)}\\ \text{i denotes a specific chemical of a series} \end{array}$$

Despite the fact that the concept of QSAR analysis was not well accepted in sixties of the last century, pharmacologist Fraser and chemist Crum-Brown from Edinburgh group wrote already in 1869: “…There cannot be any reasonable objection against a fact that a relationship between physiological effect of a compound and its chemical constitution exists….” In their paper called “On the Connection between Chemical Constitution and Physiological Action” (Crum-Brown and Fraser, 1868–1869). This concept was a subject of a famous lecture by Sir Fraser (being 31 of his age) delivered in Royal College of Physicians of Edinburgh in 1872 (Fraser 1872) (in ref. Gaddum 1962). The modern age of QSAR analysis started by works by Hansch (Hansch *et al*., 1962) and by Zahradník and Chvapil (Zahradník and Chvapil 1960). The acronym QSAR is mostly used today and started after the 1^st.^ European QSAR Symposium in Praha 1973 (Tichý 1976).

QSAR models are mathematical equations which make it possible to calculate (*in silico* methods) a magnitude of a biological, both pharmacological and toxicological, effects of chemicals using knowledge of their chemical constitution (Tichý 1983). The constitution is expressed by physicochemical properties of their molecules, by their topological indices or by quantum chemical indices, generally molecular descriptors. A demand for legislative usage of QSAR models is their statistical evaluation including new parameters of predictive toxicology (predictivity, reproducibility) and validation. The QSAR analysis requires standardization of procedures of the biological tests and profound description of their guidelines. Their most dangerous scarcity is erroneous and often bad quality of input data, both of toxicological and physicochemical nature. The biological data must be given in molar concentrations, not in weights or voumes of a sample, if results of the models are interpreted in the sense of mechanisms of action.

The molecular descriptors influencing a biological activity are of three types only: hydrophobic properties, reactivity and steric factors. The indices of these three groups cross correlated among themselves, thus looking for the most convenient one is often without any result. The experience and consequent explanation indicated partition coefficient of a chemical between n-octanol and water as the most effective. This finding is interpreted that transport of an active chemical to site of action is the activity determining step. This explanation has a physicochemical background, nevertheless reactivity and steric factors may also play a role with various weighting. This is expressed by Hansch equation (Hansch *et al*., 1962):

$$\log\text{C}={\text{k}}_{1}{\left(\log\text{P}\right)}^{2}+{\text{k}}_{2}\log\text{P}+{\text{k}}_{3}\sigma +{\text{k}}_{4}{\text{E}}_{s}+{\text{k}}_{5}$$

where C is an effective concentration causing the effect, P is partition coefficient of a chemical between n-octanol and water, σ is Hammett constant and E_s_ is Taft steric substituent constant. The parabolic form of the equation says that the relationship is generally not linear but non-linear, although mostly only the linear part of the model is experimentally found. The constants k′s are originated by statistical elaboration of a series of pairs of biological and physicochemical data of a series of chemicals analyzed.

One of the legislative usage of QSAR models will be a set of procedures making it possible to find structural alerts. They would be used to find priority groups of fragments indicating risk of a concrete effect (eye irritation, skin irritation, corrosiveness mostly discussed (OECD meeting in Utrecht 2008).

Thus, the demands o chemical safety programs and REACH may be satisfied. The QSAR models and techniques will be, finally after “tuning”, quick enough to supply data about all new chemicals, not expensive, informative as experimental models with animals.

However, nature including human beings is exposed not to single, individual chemical, but to their mixtures. And if chemicals are in a mixture the final effect can be different from just a sum of effects of individuals – there are antagonism, synergism, potentiation, inhibition,… Fortunately, the first step has been done: estimation of effect of the individuals. The further step necessarily must follow: how to estimate an effect of interaction among the chemicals being in a mixture. The behavior of chemicals in their mixture may not correspond to the predicted from data on pure chemicals. The necessity to solve the mixture toxicity has been pointed out by both scientific and regulating authorities. There have been attempts to predict the joint effects of chemical mixtures as reviewed (Tichý *et al*., 1998 among many others)

Hazard identification and its quantification is one of the starting points and essential for health risk assessment. The hazard of chemical mixtures is often assessed by a simple summation of toxic indices of individual chemicals in a mixture. This may be acceptable as long as safety limits or low-level exposures are taken into consideration. Such approach is doubtful as far as indices as EC50 or LD50ies are considered (Tichý *et al*., 2002a). The fundamental classification of joint toxic effects was introduced (eg. Hewlett and Plackett 1959, 1979). QSAR techniques were applied to estimate the toxicity of chemical mixtures to aquatic organisms (Hermens *et al*., 1985a, 1985b, Köneman 1981).

Development of this area of toxicology has become enormously increased in this decade. Papers studying possibilities of modeling mixture toxicity is not possible to list because of their number. Authors in some of them try to study involvement of hydrophobicity of compounds (Lin *et al*., 2002, 2003, Pereira *et al*., 2008 and others), some papers trying to apply QSAR modeling (a choice by chance Tichý *et al*., 1998, Conolly 2001, Altenburger *et al*., 2003, Mwense *et al*., 2006) or studying behavior of partition coefficient of chemicals being in a mixture (Rucki and Tichy 2006; Reitmajer *et al*., 2006). It is necessary to mention also extensive international projects and symposia devoted to chemical mixture toxicity (Risk Assessment of Mixtures: Development of Testable Hypotheses. Workshop of Society of Toxicology, September 2002; Anton Mixture Toxicity Workshop, Amsterdam, April 2008 and again others); or European project in Frame Program 6 of EC “ Novel Methods for Integrated Risk Assessment of Cumulative Stressors in Europe 2004–2009. And naturally many papers accompanying these activities.

Attempts to discover mechanisms behind the joint mixture toxicity using physical and organic chemistry are reasons of this interest. Actually the situation starting studies on QSAR is repeating – afford of chemical sciences to help to biological sciences. Interaction of compounds in a mixture can cause substantial changes in properties of its components. Concentration addition and independent action are two models for the evaluating of the joint activity having mechanistic support. The QSARs have been used to predict concentrations of chemicals in mixtures from joint effects and defined mixture ratios. QSAR models were developed to predict narcotic-type mixture toxicity from molecular descriptors that are calculated as composite properties according to the fractional concentrations of the mixture components as the first approximation (using ref. Altenburger *et al*., 2003).

One of possible ways, how to describe quantitatively property changes, which are dependent on mixture composition, has been initiated by chemists and statisticians: approaches of Raoult and Dalton (physicochemical text books)to physicochemical properties of chemicals in mixtures, isobolograms by Loewe and Muischnik (Loewe and Muischnik 1926) and the Finney test of additivity (Finney 1942) QCAS – Quantitative Concentration – Activity Relationships). The concept involves R-plot, ie. graphical representation of the dependence of biological activity on molar ratio (that is the R) of a mixture and its mathematical expression in a form of a function (Tichý *et al*., 1998, 2002b). One of advantages of this approach is easy recognition of additivity or non-additivity of the activity dependence on a composition of a mixture, if normalized concentration (c_N_) is used:

$$\begin{array}{l} {\text{R}}_{\text{a}}\text{=}{\text{c}}_{\text{a}}\text{/(}{\text{c}}_{\text{a}}\text{+}{\text{c}}_{\text{b}}\text{)}\\ {\text{c}}_{\text{N}}\text{=(}{\text{c}}_{\text{a}}\text{/}{{\text{c}}_{\text{a}}}^{\text{o}}\text{)/(}{\text{c}}_{\text{b}}\text{/}{{\text{c}}_{\text{b}}}^{\text{o}}\text{)} \end{array}$$

where c_a_ and c_b_ are concentrations of chemicals in a binary mixture, c_a_^o^ and c_b_^o^ concentrations of single components of a mixture, a and b, causing the same activity (EC50). In terms of biological activities a normalized EC50 (EC50_N_) is:

$$\text{EC5}{\text{0}}_{\text{N}}\text{ = [EC50 (a + b)] / [}{\text{R}}_{\text{aN}}\text{.EC50a + }{\text{R}}_{\text{bN}}\text{.EC50b]}$$

where EC50(a + b) is effective concentration of binary mixture, EC50a and EC50b effective concentrations of pure compounds a and b, R_aN_ and R_bN_ normalized molar ratios of compounds a or b in a binary mixture. The EC50_N_ thus serves as a measure of additivity. In the R-plot, the ends of x-axes are formed by EC50ies of pure compounds divided by the same, EC50ies of the compounds, and thus reaching values 1.

Effects of compounds a and b are additive ifa replacement of some part of one compound in a mixture by an equipotent part of other compound does not change the effectivity of the mixture (concentration addition). The additivity is, thus, defined by the relation:

$${\text{[EC50(a)}}_{\text{mix}}{\text{/ EC50(a)] + [EC50(b)}}_{\text{mix}}\text{/ EC50(b)] = 1}$$

If not equal 1, the effects are not additive. In the R-plot it is a horizontal line y = 1, if additive. The curve can be then describe by a polynomical function, upto the fifth order would be sufficiently exact (Tichý *et al*., 1998, Tichý *et al*., 2005).
